# Identification of a rare SEPT9 variant in a family with autosomal dominant Charcot-Marie-Tooth disease

**DOI:** 10.1186/s12881-020-0984-7

**Published:** 2020-03-02

**Authors:** Gerrit M. Grosse, Christine Bauer, Bruno Kopp, Christoph Schrader, Alma Osmanovic

**Affiliations:** 1 Department of Neurology, Hannover Medical School, Carl-Neuberg-Str. 1, 30625 Hannover, Germany; 2 Center for Genomics and Transcriptomics (CeGaT GmbH), Tübingen, Germany

**Keywords:** Charcot-Marie-Tooth, Next generation sequencing, SEPT9, Septin, Inherited neuropathy

## Abstract

**Background:**

Charcot-Marie-Tooth disease (CMT) is one of the most commonly inherited neurological disorders. A growing number of genes, involved in glial and neuronal functions, have been associated with different subtypes of CMT leading to improved diagnostics and understanding of pathophysiological mechanisms. However, some patients and families remain genetically unsolved.

**Methods:**

We report on a German family including four affected members over three generations with a CMT phenotype accompanied by cognitive deficits, predominantly with regard to visual abilities and episodic memory.

**Results:**

A comprehensive clinical characterization followed by a sequential diagnostic approach disclosed a heterozygous rare SEPT9 missense variant c.1406 T > C, p.(Val469Ala), that segregates with disease. SEPT9 has been linked to various intracellular functions, such as cytokinesis and membrane trafficking. Interestingly, SEPT9-mutations are known to cause hereditary neuralgic amyotrophy (HNA), a recurrent focal peripheral neuropathy.

**Conclusion:**

We, for the first time, present a SEPT9 variant associated to a CMT phenotype and suggest SEPT9 as new sufficient candidate gene in CMT.

## Background

Charcot-Marie-Tooth disease (CMT), also known as hereditary motor and sensory neuropathy, comprises a genetically and clinically heterogeneous group of genetic disorders that mainly affect peripheral nerves. CMT is clinically characterized by distal muscle weakness and atrophy, sensory loss, depressed tendon reflexes, and foot deformity. Growing knowledge about additional clinical features associated with CMT makes it a syndromic disease [1]. Most commonly, CMT is neurophysiologically subdivided into a demyelinating (CMT1) and axonal (CMT2) form depending on whether nerve conduction velocity (NCV) is below or above 38 m/s [2]. Up to 60–70% of demyelinating CMT cases are caused by duplication of peripheral myelin protein 22 gene (PMP22). Together with mutations in myelin protein zero (MPZ), gap junction protein beta 1 (GJB1), and mitofusin 2 (MFN2), over half of CMT cases can be explained [3]. So far, more than 80 CMT-genes have been identified mainly through linkage analysis and next generation sequencing (NGS) studies. Most of the identified genes related to CMT are encoded in nerval structures like axons or myelin and functionally involved in cytoskeleton network, membrane trafficking and myelination. Therefore, genes involved in cellular processes and biological functions concerning Schwann cells and axonal transport processes, are of high interest in so far genetically unsolved CMT patients [4].

In this study we performed a sequential approach for genetic testing, including a NGS multi gene panel with known CMT-genes and promising candidate genes, in a clinically well characterised CMT1-family. We identified a rare heterozygous missense variant in the SEPT9 gene, a gene previously described as a cause of hereditary neuralgic amyotrophy (HNA) when heterogeneously mutated [5]. SEPT9 is part of the SEPT3-group and encodes the ubiquitously expressed septin 9 protein, which belongs to the conserved septin family of GTPases involved in various cellular processes such as motility and cytokinesis [6]. Interestingly, septin 9 interacts with the cytoskeleton including microtubules and actin and thereby promotes asymmetric neurite outgrowth [7]. Here, we genetically and clinically present an association of a SEPT9 gene alteration to a distinct CMT phenotype and discuss potential effects on protein level.

## Methods

### Subjects

**Fig. 1:**
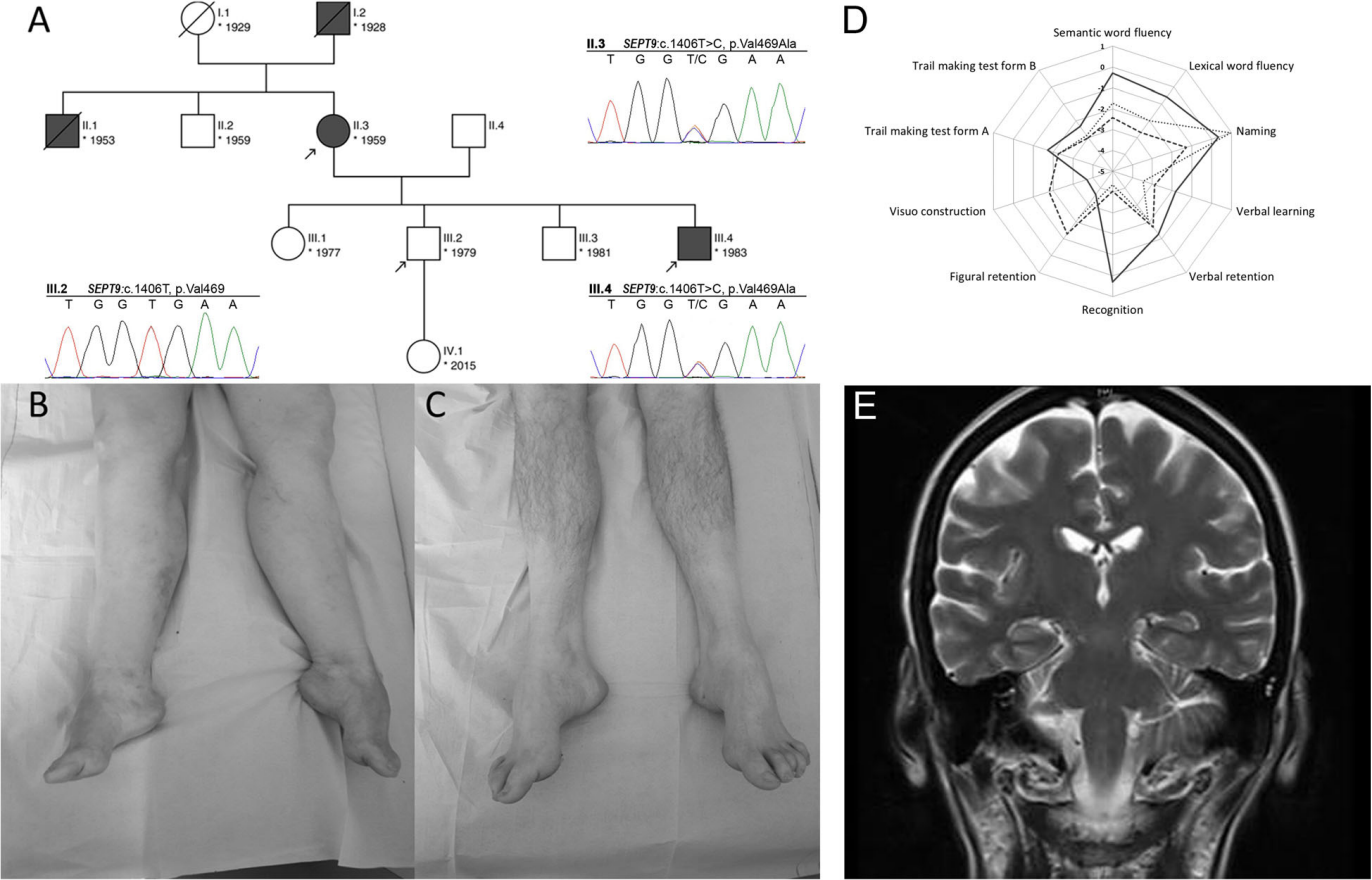
**a** Pedigree chart of the family, arrows indicate subjects enrolled in this study. Electropherograms obtained by Sanger sequencing demonstrate the heterozygous missense variant SEPT9:c.1406 T > C, p.Val469Ala in subject II.3 and III.4, which was not detected in the healthy subject III.2. **b** Lower extremities of patient II.3; **c** lower extremities of patient III.4. **d** Results from neuropsychological assessment using the CERADplus test battery in patients II.3 and III.4. Dashed line: II.3 (2014); dotted line: II.3 (2015); solid line: III.4 (2016). z < − 2.0: severely affected; − 1.99 < z < − 1.50: mildly affected; − 1.49 < z < − 1.0: marginal affected; z ≥ − 1.0: unaffected. Visuo constructive abilities from II.3 could not be measured in 2015 due to severe motor dysfunctions. **e** MRI of patient II.3, T2 coronar

We investigated a northern German CMT1-family with four affected members over three generations. The local ethics committee and all participants consented to the analysis. Three individuals could be enrolled (Fig. 1a).

Index case II.3 underwent an extensive work-up including a thorough clinical evaluation, physical examination, nerve conduction studies, cranial magnetic resonance imaging (MRI), lumbar puncture, routine and specified blood tests. Cognition was assessed using the Montreal Cognitive Assessment (MOCA) [8] and additionally using the “Consortium to Establish a Registry for Alzheimer’s Disease” battery (CERADplus) [9].

Healthy subject III.2 and his affected brother III.4 were evaluated subsequently by neurological examinations, nerve conduction studies and neuropsychological testing, as described above. Individual III.4 was further assessed by cranial MRI.

### Genetic testing

Deletions and Duplications in PMP22, MFN2 and MPZ were analysed by Multiplex ligation-dependent probe amplification (MLPA), performed on extracted DNA obtained from index patient II.3 whole blood sample. Subsequently, NGS was carried out in this subject at the CeGat-Institute, Tübingen, Germany, with a multi gene panel-based strategy including following genes known to be associated with or linked to hereditary neuropathy: AARS, ABHD12, AIFM1, ARHGEF10, ATL1, ATL3, BSCL2, C10ORF2, C12ORF65, CTDP1, DCAF8, DHTKD1, DNM2, DNMT1, DST, DYNC1H1, EGR2, FAM134B, FBLN5, FGD4, FIG4, GAN, GARS, GDAP1, GJB1, GNB4, HADHA, HADHB, HARS, HINT1, HK1, HOXD10, HSPB1, HSPB8, IFRD1, IGHMBP2, IKBKAP, INF2, KARS, KIF1A, KIF1B, KIF5A, LITAF, LMNA, LRSAM1, MARS, MED25, MFN2, MPZ, MTMR14, MTMR2, NDRG1, NEFL, NGF, NTRK1, OPA1, OPA2, PDHA1, PDK3, PLEKHG5, PMP22, POLG, PRPS1, PRX, RAB7A, REEP1, SBF1, SBF2, SCN9A, SEPT9, SH3TC2, SLC12A6, SOX10, SPTLC1, SPTLC2, SURF1, TFG, TRIM2, TRPV4, TTR, TYMP, WNK1, YARS. Only variants affecting coding exons or canonical splice sites were selected. Synonymous variants were filtered out. Since the CMT1-family in our study expose an autosomal dominant inheritance pattern only rare variants with a minor allele frequency (MAF) of < 0.1% in the European population, according to the global databases, ExAC Browser (http://exac.broadinstitute.org/) and gnomAD (https://gnomad.broadinstitute.org/) as well as an in-house database of the CeGat Institute, Tübingen, Germany, were considered. In silico predictions of the longest SEPT9 transcript ENST00000427177.6 (NM_006640) were taken from Alamut Visual software version 2.8® (Interactive Biosoftware) which combines different prediction programs (SIFT, Mutation Taster and PolyPhen) (Table 1). For further variant characterisation the Combined Annotation Dependent Depletion (CADD) score [11] and the American College of Medical Genetics (ACMG) criteria [10] were used. We verified and evaluated our identified variant for co-segregation with the phenotype in individuals III.2 and III.4 by Sanger sequencing (Fig. 1a).

**Table 1:** Rare heterozygous non-silent variant in the SEPT9 gene predicted to be deleterious identified in a german family with CMT

Sample	Chromosomal position (GRCh37/hg19)	Exon	Nucleotide change	Amino-acid change	SNP number	MAF	Prediction			ACMG
							SIFT	PolyPhen-2	Mutation-Taster	
II.3 III.4	17:75488782	8	c.1406 T > C	p.(Val469Ala)	rs376712636	0.0006837	Deleterious	Benign	disease causing	Class 3 (VUS)
III.2	/	/	/	/	/	/	/	/	/	/

*Abbreviations*: *ACMG* American College of Medical Genetics and Genomics according to Richards et al. 2015 [10], Genetics in Medcine, *MAF* minor allele frequency in European (non-Finnish) population according to Exome Aggregation Consortium Browser (Beta); Reference sequence used: NM_006640. *VUS* variant of uncertain significance

## Results

### Clinical features

The index patient II.3, a then 55-years-old woman with a past medical history of breast cancer and B-cell non-Hodgkin lymphoma, presented with progressive muscular weakness and moderate sensory deficits, with an estimated symptom onset at 40 years. Neurological examination revealed bilateral pes cavus (Fig. 1b), intermittent postural tremor of the right upper extremity, distal muscle weakness, diminished deep tendon reflexes and hypesthesia of the lower extremities as well as severe gait ataxia. Furthermore, she complained of forgetfulness, impaired concentration and disorientation, first noted at the age of 53 years. Her nerve conduction studies were consistent with a primarily demyelinating motor and sensory polyneuropathy (Table 2). The initial neuropsychological assessment of II.3 yielded a MOCA score of 20/30, indicating the presence of cognitive deficits. The more comprehensive assessment with the CERADplus neuropsychological test battery revealed deficits mainly in visual constructive abilities (*z* = − 1.8), in verbal learning and memory (verbal learning, *z* = − 2.88; verbal recognition, *z* = − 4.05), and on both Trail Making Tests (Form A, *z* = − 2.25; Form B, *z* = − 3.01) (see also Fig. 1d; dashed line). Her estimated premorbid intelligence, as determined using a multiple-choice vocabulary intelligence test (MWT-B) [12] (raw score: 21), fell within the range of age-adjusted normal values, rendering it unlikely that the observed cognitive deficits were due to premorbid mental retardation. Cranial MRI revealed neither general nor regional atrophy nor white matter lesions (Fig. 1e). Blood tests and cerebrospinal fluid analysis did not show any pathological results, including normal results for Tau, pTau and Amyloid-β.

**Table 2:** Clinical and diagnostic characteristics of two *SEPT9* variant carriers (II.3 and III.4) compared to an unaffected family member (II.2)

Subject	Sex	Genetics	Clinical features						Nerve conduction studies							
		Nucleotide Change	Age at Onset	Symptom at Onset	Amyotrophy	Weakness	Sensory loss	DTR	Ulnar nerve				Peroneal nerve		Sural nerve	
			(y)		UL/LL	UL/LL	UL/LL	UL/LL	CMAP	DL/MNCV	SNAP	SCV	CMAP	DL/MNCV	SNAP	SCV
II.3†	F	*SEPT9*, c.1406 T > C	40^#^	ataxia, hyp-aesthesia	++/++	+/+	+/+	1/0	5.3	4.6/37	NR	31	1.3	7.2/30	NR	NR
III.2	M	–	N/A	N/A	−/−	−/−	−/−	2/2	8.9	2.9/62	17.8	50	10.1	3.8/50	17.8	47
III.4	M	*SEPT9*, c.1406 T > C	20^#^	ataxia, hyp-aesthesia	++/++	−/−	−/+	1/1	6.0	5.2/21	NR	27	NR	NR	NR	NR

Index case (†), *M* male, *F* female, *LL* lower limbs, *UL* upper limbs; amyotrophy (none, −; slight, +; moderate, ++; severe, +++); weakness (none, −; slight, +; moderate, ++; severe, +++); DTR, deep tendon reflexes, (areflexia 0, hyporeflexia 1, normal 2), *NA* not applicable. (^#^) approximately, Age at onset in years (y) *CMAP* compound muscle action potential [mV], *SNAP* sensory nerve action potential [μV], *DL* distal latency [ms], *MNCV* motor nerve conduction velocity [m/s], *SCV* sensory conduction velocity [m/s], *NR* no response. – none. Chromosomal position (GRCh37/hg19) 17:75488782, Reference sequence used: NM_006640.4

At follow-up 2 years later, the patient was dependent of walking aids and of legal guardianship for financial affairs. The initially observed pattern of cognitive deficits proved remarkably stable (see Fig. 1d; dotted line).

A second family subject, II.3 youngest son (III.4, *1983), exhibit almost similar symptoms: bilateral pes cavus (Fig. 1c), intermittent postural tremor of the left upper extremity, hypesthesia of the lower extremities and severe gait ataxia, while demonstrating an earlier age at onset approximately with 20 years, and a more severe primarily demyelinating sensory and motor neuropathy in nerve conduction studies (Table 2). Neuropsychological assessment of III.4 yielded a MOCA score of 23/30, and CERADplus scores indicating deficits in visual constructive abilities (*z* = − 3.71), and on the Trail Making Tests (Form A, *z* = − 1.71; Form B, *z* = − 2.34) (see also Fig. 1d). Like in his mother, his cranial MRI revealed no specific abnormality. His past medical history included substance abuse of tetrahydrocannabinol (THC).

Subject III.2, (*1979) second child of our index patient, was phenotypically healthy. No subclinical pathologies were found in either nerve conduction studies (Table 2) nor in the neuropsychological assessment (he scored 29/30 on the MOCA). Past medical history was unremarkable, besides past consumption of THC and temporary depressive disorder.

Unfortunately, additional family members were not available for clinical or genetic assessment. The family history, however, revealed more probably identically affected subjects: The index patient’s father (I.2; *1928; ✝1975) as well as one older brother (II.1; *1953; ✝1992) had suffered from polyneuropathy with bilateral pes cavus and cognitive deficits; Remarkably, the index patient’s twin brother (II.2; *1959) was reported to be phenotypically healthy. The index patient had two more children, one daughter (III.1; *1977), who had been diagnosed with early childhood cerebral palsy, and one further son (III.3; *1981), who was said to have no symptoms or signs of neuropathy.

### Genetic analysis

**Fig. 2:**
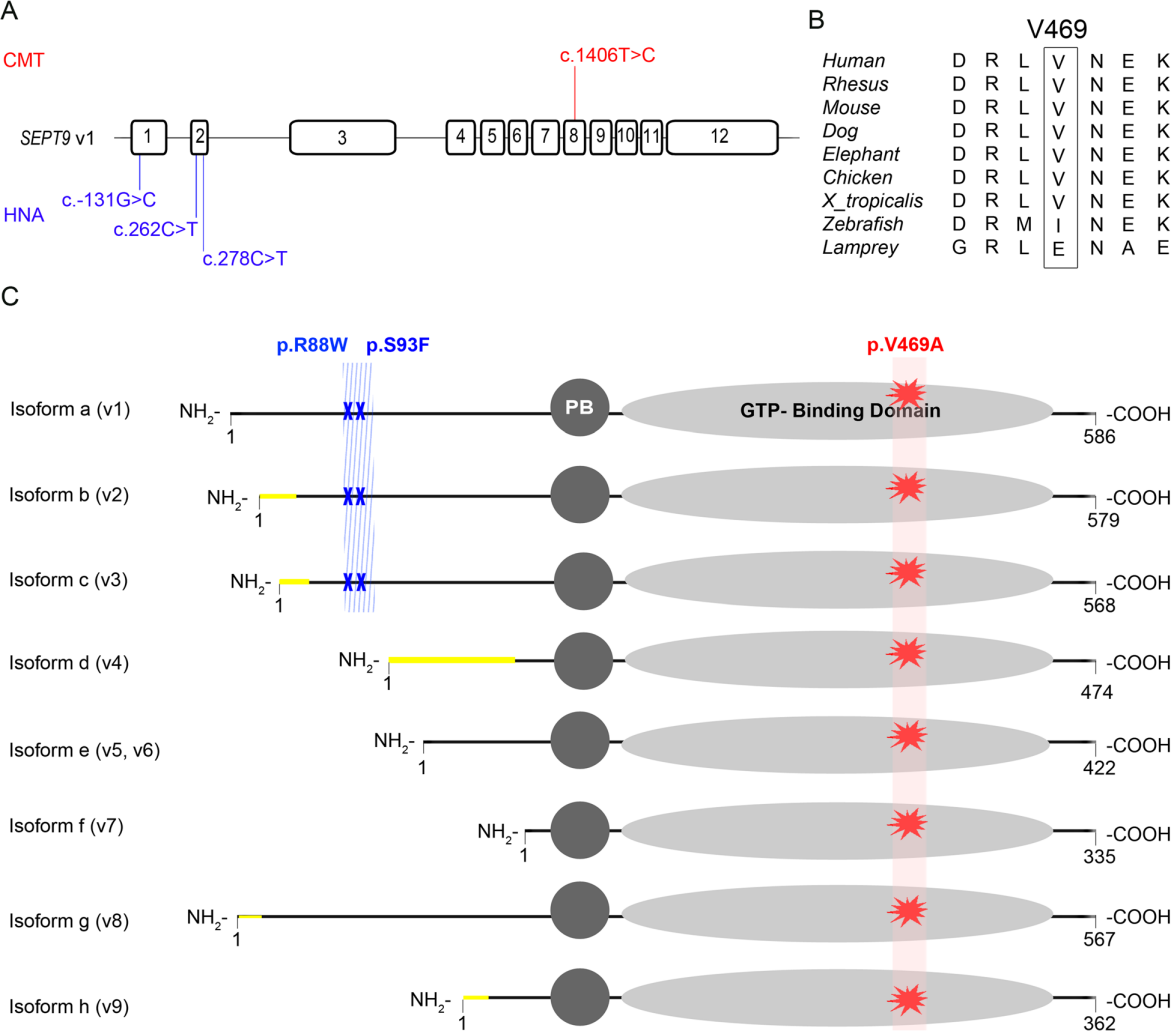
**a** Schematic representation of the SEPT9 gene with the identified heterozygous variant c.1406 T > C in Exon 8 in CMT compared to HNA-associated variants in the 5’UTR, Exon 1 and Exon 2. (not drawn to scale). ENST00000427177.6. **b** Evolutionary conservation of Valin at position 469 in septin9. **c** Schematic domain structure of SEPT9 isoforms and their functional domains. Highlighted in red identified variant in this study, in blue known HNA linked variants. PB: polybasic region

Deletions or Duplications in PMP22, MFN2 and MPZ were excluded in our index case II.3. By targeted NGS and variant prioritization we identified a heterozygous single nucleotide change c.1406 T > C in exon 8 of the SEPT9 gene (GRCh37/hg19 chr17:75488782) resulting in the amino acid change p.(Val469Ala) which - to our best knowledge – have not been reported in clinical context so far (Fig. 2a). According to the databases mentioned above (gnomAD and ExAC), MAF of the identified SEPT9 variant was very low (0.02%) in the European (non-Finnish) population. The resulting p.(Val469Ala) substitution occurred at an evolutionarily conserved amino acid residue (Fig. 2b). In silico prediction analysis revealed pathogenicity in 2 out of 3 prediction tools (Table 1). The CADD score was 22.7 (CADD GRCh37-v1.4), indicating that the variant belongs to the top 1% of deleterious variants in the human genome [11]. In segregation analysis (subject III.2 and III.4) we found co-segregation of the distinct CMT1 phenotype, here III.4, with the SEPT9 variant. The variant was classified as “uncertain significance” (class 3, evidence of pathogenicity: PP1, PP2, PP3, PP4) based on ACMG guidelines [10].

## Discussion

In this study, we used a targeted NGS approach to investigate the genetic cause of a distinct CMT1-phenotype in a German family. We identified a novel rare heterozygous SEPT9 variant, c.1406 T > C, (p.Val469Ala) in an evolutionary conserved region, predicted to be pathogenic. The variant was confirmed to co-segregate with the CMT-phenotype within the family.

Heterozygous SEPT9 mutations have been linked to hereditary neuralgic amyotrophy (HNA), a rare autosomal dominant recurrent focal neuropathy, clinically characterized by acute episodes of brachial plexus neuropathy with muscle weakness and atrophy, preceded by severe pain [5]. Interestingly, phenotypic heterogeneity in HNA has been well documented, such as persisting non-recurrent neuropathy symptoms and involvement of the lumbosacral plexus [13]. Besides this, additional clinical features, mainly dysmorphic symptoms and even bilateral pes cavus, have been found in individuals with HNA [14], which is a hallmark clinical symptom of CMT neuropathy. Both affected individuals in this study demonstrate this clinical feature (Fig. 1b/c).

Electrophysiological studies in HNA show predominantly axonal damages, however demyelination patterns, largely present in our family, have been detected in HNA likewise [15]. SEPT9 is highly expressed in glia cells, including Schwann cells which form the myelin sheet in neuronal tissues. Remarkably, a previous histological study demonstrated minor onion bulb formations, a neuropathological hallmark of CMT1, in an HNA patient, suggestive of Schwann cell alterations [16].

The ubiquitously expressed septin 9 (SEPT9) is one of the 13 members of septins, a highly conserved family of GTP binding proteins that have been linked to a broad spectrum of cellular functions. Septins interact with the cytoskeleton, including actin, and function in processes such as cytokinesis, motility, and cell polarity. Other functions include angiogenesis and bacterial autophagy (for review see [6]). SEPT9 knockout in mice results in embryonic lethality [17]. Septins form oligomeric complexes, which can assemble into higher ordered structures such as filaments and rings. A well-known septin complex consists of alternating SEPT2/6/7/9-units [18].

The SEPT9 gene gives rise to at least eight different transcript variants generated by extensive alternative splicing of the N-terminal domain, which may have tissue-specific expression (Fig. 2c). However, all transcript variants maintain a highly conserved central region containing the polybasic domain (PB) preceding a GTP-binding domain (G-domain) [19].

Interestingly, previously identified missense mutations in HNA patients – namely c. 262C > T, p.(Arg88Trp); c.278C > T, p.(Ser93Phe); c.-131G > C (in 5’UTR) affect the N-terminal region. Hence, only a minority of septin9 isoforms are altered by these mutations [19] (Fig. 2c). In contrast, our identified variant c.1406 T > C, p.(Val469Ala), located in the C-terminal region, occurs in all known transcripts of SEPT9 (Fig. 2c). Therefore, a far more severe phenotype due to the involvement of multiple tissues, for instance the central nervous system (CNS), can be presumed.

To note, known genetic variants associated with cognitive impairment were not excluded in this family. However, due to similar cognitive deficits in the here identified SEPT9-variant carriers and the positive family history, cognitive deficits may be an additional clinical feature in the SEPT9-phenotype spectrum. Of interest, alterations of other members of the septin family have been associated with neurodegenerative disorders [20]: SEPT1–4 have been linked to Alzheimer’s disease while for SEPT5 an association to Parkinson’s disease has been reported [20]. Thus, one might speculate that the SEPT9 alteration could be associated with the cognitive changes in our current study. This hypothesis is further supported by the characteristic feature of septins to form oligomeric complexes [21]. Septin-septin interactions are formed by oligomerization of two neighbouring G-domains. The identified Substitution p.(Val469Ala) in this study is situated within the functionally relevant (G-domain) (Fig. 2c). By an alanine-for-valine substitution in this binding domain, essential protein-protein interactions and functions may be disturbed, particularly as valine unlike alanine contains two non-hydrogen substituents attached to their c-beta carbon [22]. Due to the location of p.(Val469Ala) in a central domain of the protein and the potential demolishing impact on oligomerisation we suppose a rather loss of function effect. Further studies are needed to confirm this hypothesis.

## Conclusions

We identified a novel rare heterozygous SEPT9 gene variant in a family demonstrating a distinct CMT1 phenotype with overlapping clinical features to SEPT9 associated HNA and suggest a potential relevant functional implication on protein level. Thus, SEPT9 alterations may be considered as a genetic risk-factor in CMT1. However, future clinical and functional studies are needed to elucidate the pathogenic relevance.

## Data Availability

The identified variant in this study has been submitted to the ClinVar database at NCBI archives (ID: SCV001156521; https://www.ncbi.nlm.nih.gov/clinvar/). All data generated or analysed during this study are included in this published article.

## Abbreviations

ACMG: American College of Medical Genetics
CADD: Combined Annotation Dependent Depletion
CERADplus: Consortium to Establish a Registry for Alzheimer’s Disease battery
CMAP: Compound muscle action potential
CMT: Charcot-Marie-Tooth disease
DL: Distal latency
DTR: Deep tendon reflexes
F: Female
HMSN: Hereditary motor and sensory neuropathy
HNA1: Hereditary neuralgic amyotrophy 1
LL: Lower limbs
M: Male
MNCV: Motor nerve conduction velocity
MOCA: Montreal cognitive assessment
MRI: Magnetic resonance imaging
NA: Not applicable
NR: No response
PB: Polybasic region
SCV: Sensory conduction velocity
SNAP: Sensory nerve action potential
THC: Tetrahydrocannabinol
UL: Upper limbs
